# The patient-level economic burden of COPD in China: a systematic review of determinants and consequences

**DOI:** 10.3389/fpubh.2026.1850249

**Published:** 2026-06-29

**Authors:** Jian Tian, Yue Zhao, Zhi Qu, Cheng Wang, Rongsha Yang

**Affiliations:** 1 Maternity and Child-care Hospital of Gansu Province, Gansu Provincial Central Hospital, Lanzhou, China; 2 Hannover Medical School, Institute for Epidemiology, Social Medicine and Health Systems Research, Hannover, Germany; 3 Center for Health Economics Research Hannover (CHERH), Hannover, Germany; 4 Gansu Provincial Hospital of Traditional Chinese Medicine, Lanzhou, China

**Keywords:** catastrophic health expenditure, China, COPD, economic burden, health policy, out-of-pocket expenditure

## Abstract

**Background:**

Chronic obstructive pulmonary disease (COPD) imposes a substantial patient-level economic burden in China, including direct medical expenditure, indirect productivity losses, out-of-pocket payments, and catastrophic health expenditure. However, evidence on the magnitude, determinants, and consequences of this burden remains fragmented across regions, healthcare settings, and study designs.

**Methods:**

We conducted a systematic review of empirical studies reporting patient-level economic burden related to COPD in China. Searches were conducted in international and Chinese databases from inception to December 31, 2025. Observational and quasi-experimental studies were eligible if they reported direct costs, indirect costs, catastrophic health expenditure, out-of-pocket expenditure, financial hardship, or policy-related economic outcomes. Due to substantial heterogeneity in study designs and outcome measures, findings were synthesized narratively.

**Results:**

Twenty-five studies published between 2002 and 2025 were included. Direct medical costs, particularly hospitalization expenditure related to acute exacerbations of COPD (AECOPD), were the most frequently reported component of burden. Severe exacerbations, greater disease severity, longer hospital stay, intensive care use, mechanical ventilation, comorbidities, poor medication adherence, and anxiety/depression were consistently associated with higher healthcare costs. Indirect costs from productivity loss and work impairment frequently equaled or exceeded direct medical expenditure. Socioeconomic inequality was also evident, with lower-income and rural households experiencing disproportionately higher catastrophic health expenditure and out-of-pocket burden. Policy-related studies suggested that integrated payment reform and comprehensive hospital reform were associated with fewer avoidable readmissions and lower patient out-of-pocket costs, although causal interpretation remains limited by study design and potential confounding.

**Conclusion:**

COPD imposes a substantial and multidimensional patient-level economic burden in China. The evidence identifies AECOPD-related hospitalization as a major cost driver and highlights the importance of indirect costs, socioeconomic inequality, and healthcare financing structures. Strategies aimed at preventing severe exacerbations, improving long-term disease management, expanding outpatient reimbursement, and strengthening financial protection for vulnerable populations may help mitigate COPD-related economic burden. Further longitudinal and policy-evaluation studies using standardized economic outcome measures are needed.

**Systematic review registration:**

https://www.crd.york.ac.uk/PROSPERO/view/CRD420251043025.

## 1 Background

Chronic obstructive pulmonary disease (COPD) is a leading cause of morbidity and mortality worldwide and represents a major public health challenge, particularly in low- and middle-income countries (LMICs) (1–3). The World Health Organization estimates that over 90% of COPD-related deaths occur in LMICs, where populations experience high exposure to risk factors alongside limited access to effective long-term care (4, 5). China bears approximately 25% of the global COPD burden, with national surveys reporting a COPD prevalence of 13.6% among adults aged 40 years and older (6–9). The disease is especially prevalent in rural and less-developed regions, where healthcare access and reimbursement resources remain comparatively limited (10).

For individuals and their families, a diagnosis of COPD is associated with a significant and sustained patient-level economic burden. This burden includes direct medical costs related to recurrent hospitalizations, long-term oxygen therapy, inhaled maintenance medications, and regular outpatient care (11). Indirect costs, including productivity loss, disability-related income reduction, and caregiver burden, may further exacerbate household financial strain. As COPD is a chronic and progressive condition, these costs often accumulate over time, generating persistent financial strain that can escalate into catastrophic health expenditure for households. The extent of this economic burden is strongly influenced by healthcare financing structures, insurance reimbursement policies, and access to chronic disease management services. In China, regional disparities in healthcare financing and insurance reimbursement may further exacerbate the economic impact of COPD on affected households (12). Although public insurance schemes have expanded population coverage, high out-of-pocket (OOP) payments for outpatient medications and long-term care remain common, particularly in rural areas (13). Hospitalizations for acute exacerbations represent a major driver of medical impoverishment, and a considerable portion of these events may be preventable with optimized disease management (14). Consequently, inadequate disease control may contribute not only to poorer clinical outcomes but also to escalating household financial burden.

Beyond material costs, the patient-level economic burden of COPD has important psychosocial consequences. Patients and their families commonly report anxiety and depression related to the long-term affordability of care and the economic consequences of the disease (15). In resource-limited households, this financial pressure may force trade-offs between essential healthcare and basic living needs.

Despite growing recognition of these challenges, evidence regarding the patient-level economic burden of COPD in China remains fragmented across regions, healthcare settings, and study designs. While risk factors such as disease severity and inadequate insurance are known to contribute to financial hardship, the relationships among direct medical costs, indirect economic burden, catastrophic health expenditure, healthcare financing factors, and associated patient outcomes have not been systematically synthesized. In light of these gaps, the present study systematically reviews and synthesizes evidence from China on the patient-level economic burden of COPD. Specifically, it aims to identify determinants of this burden and examine its associations with clinical, psychosocial, and economic outcomes, with the goal of informing healthcare policy and guiding targeted healthcare and policy interventions to mitigate the financial impact of COPD on patients and their families.

## 2 Methods

### 2.1 Protocol and registration

This systematic review was conducted and reported in accordance with the Preferred Reporting Items for Systematic Reviews and Meta-Analyses 2020 (PRISMA 2020) statement (16). The review protocol was prospectively registered with the International Prospective Register of Systematic Reviews (PROSPERO; Registration No. CRD420251043025).

### 2.2 Literature search

A comprehensive search strategy was developed by two authors and refined by the full research team. Systematic searches were conducted in PubMed, Embase, Web of Science Core Collection, and the Chinese databases CNKI and WanFang from database inception to December 31, 2025. Chinese databases were included to improve retrieval of region-specific literature that may not be indexed in international databases.

The search strategy combined controlled vocabulary terms (e.g., MeSH and Emtree terms) and free-text keywords across three major concepts: (1) COPD; (2) economic burden, healthcare expenditure, financial hardship, and insurance-related factors; and (3) China. Search strategies were adapted appropriately for the syntax and indexing system of each database. The Embase search used Emtree controlled vocabulary terms combined with free-text keywords, while topic-field searches were conducted in CNKI and WanFang using Chinese keywords related to COPD, economic burden, healthcare expenditure, insurance reimbursement, and China. Complete database-specific search strategies are provided in Supplementary file 1.

To supplement database retrieval and enhance search sensitivity, Google Scholar was searched using combinations of keywords related to COPD, economic burden, healthcare costs, and China. The first 200 records sorted by relevance were screened for potentially eligible studies. Reference lists of all included studies and relevant reviews were manually screened to identify additional eligible articles. All retrieved records were imported into reference management software, and duplicate records were removed prior to screening.

### 2.3 Eligibility criteria

Studies were included in this review if they met the following pre-specified criteria:

- Population: Included adult patients (≥18 years) with physician-diagnosed COPD or COPD identified through medical records, insurance claims, registries, or validated diagnostic criteria.
- Setting: Conducted in mainland China.
- Outcomes: Reported data on patient-level economic burden, including direct medical costs (e.g., out-of-pocket expenditures and hospitalization costs), indirect costs (e.g., productivity loss or income reduction), catastrophic health expenditure, financial hardship, or other economic outcomes related to COPD.
- Study design: Included empirical quantitative studies with observational or quasi-experimental designs, such as cross-sectional, cohort, registry-based, nonrandomized comparative, and interrupted time-series studies, provided they reported patient-level economic outcomes related to COPD.

Studies published in English or Chinese were eligible for inclusion. Studies conducted outside mainland China were excluded because differences in healthcare financing and insurance systems may substantially influence patient-level economic burden. Review articles, editorials, commentaries, protocols, conference abstracts without sufficient data, qualitative studies, animal studies, and other non-original research publications were excluded. Studies that did not report explicit measures of patient-level economic burden relevant to the review objectives were also excluded.

### 2.4 Study selection and data extraction

All retrieved records were imported into reference management software Citavi 6, and duplicate records were removed prior to screening. Two reviewers independently screened titles and abstracts to identify potentially eligible studies. Potentially eligible articles subsequently underwent full-text review independently by two reviewers according to the predefined inclusion and exclusion criteria. Disagreements at any stage of the selection process were resolved through discussion and consensus or, when necessary, consultation with a third senior reviewer.

A standardized data extraction form was developed and piloted prior to formal extraction. Two reviewers independently extracted data from each included study, including study design, setting, sample size, participant characteristics, COPD-related clinical measures, definitions and measures of economic burden, healthcare financing or reimbursement characteristics, and key findings related to direct medical costs, indirect economic burden, catastrophic health expenditure, and associated clinical or psychosocial outcomes.

### 2.5 Methodological quality and risk of bias assessment

Methodological quality and risk-of-bias assessments were conducted independently by two reviewers, with disagreements resolved through discussion and consensus. For observational cohort, retrospective database, and cross-sectional studies, the NIH Quality Assessment Tool for Observational Cohort and Cross-Sectional Studies was used. Studies were categorized as good, fair, or poor quality according to NIH guidance based on the number and relevance of applicable criteria fulfilled. Items judged as “not applicable” were excluded from the overall assessment because certain criteria, such as participation rate, blinding, or loss to follow-up, were not relevant to administrative database studies or routinely collected economic outcomes.

For nonrandomized comparative studies and interrupted time-series analyses, additional design-specific risk-of-bias considerations adapted from ROBINS-I and Cochrane Effective Practice and Organization of Care (EPOC) guidance were applied. These considerations included potential confounding, group comparability, pre-intervention trends, concurrent policy or system changes, autocorrelation or seasonality, and reliability of outcome measurement. These assessments informed the narrative interpretation of findings, particularly for studies evaluating healthcare financing, reimbursement policies, or healthcare utilization interventions (17).

### 2.6 Data synthesis

Due to substantial heterogeneity in study designs, healthcare settings, economic outcome definitions, and methods used to measure patient-level economic burden, quantitative meta-analysis was considered inappropriate. Therefore, findings were synthesized narratively. The synthesis was organized thematically according to the reported magnitude and dimensions of economic burden, including direct medical costs, indirect economic burden, catastrophic health expenditure, and psychosocial consequences, as well as factors associated with increased financial burden. Findings related to healthcare financing, reimbursement policies, and healthcare utilization were also summarized where applicable. The interpretation of findings considered the methodological quality and risk-of-bias assessments of the included studies.

## 3 Results

### 3.1 Study selection and characteristics

**Figure 1:**
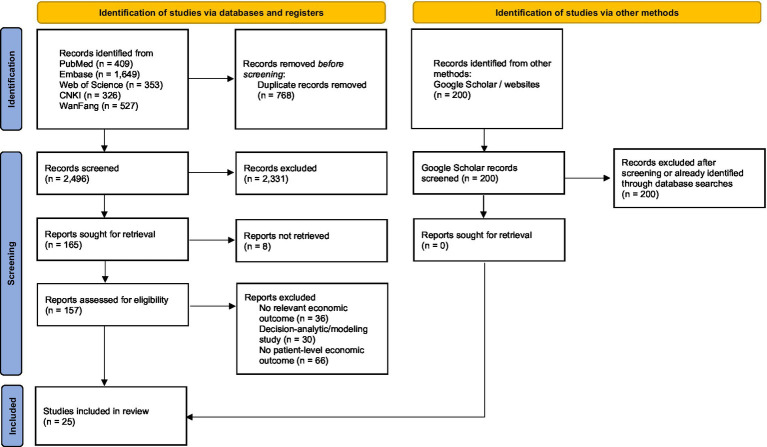
PRISMA 2020 flow diagram of study selection. Flow diagram showing the identification, screening, eligibility assessment, and inclusion of studies in this systematic review. The first 200 Google Scholar records were screened as supplementary sources; no additional eligible full-text reports were identified beyond the database searches.

Database searches identified 3,264 records. After removal of 768 duplicates, 2,496 records were screened. In addition, the first 200 Google Scholar records were screened as supplementary sources, but no additional eligible studies were identified. After full-text assessment, 25 studies met the inclusion criteria and were included in the narrative synthesis (18–42). The study selection process is summarized in the PRISMA flow diagram (Figure 1).

The characteristics of the included studies are summarized in Table 1. The included studies were published between 2002 and 2025 and demonstrated substantial methodological heterogeneity. Included study designs comprised cross-sectional surveys, retrospective observational studies, retrospective cohort studies, prospective cohort studies, nonrandomized comparative studies, and interrupted time-series analyses. Sample sizes ranged from 85 participants in a single-center inpatient survey to more than 100,000 patients in multicenter hospital database studies (21, 29). Data sources included hospital information systems, insurance claims databases, electronic health records, national surveys, and community-based investigations conducted across both urban and rural regions of China (22, 30, 39, 40).

**Table 1:** Characteristics of included studies.

First author, year	Study design	Setting/data source	Sample size	Age/male sex
Ren, 2002 (18)	Prospective follow-up survey	Four communities, Sichuan	205 patients	Age not specified; 51.2% male
Chen, 2008 (19)	Retrospective multicenter study	Four hospitals, Beijing	439 patients	73.4 years; 67.0% male
Guo, 2010 (20)	Cross-sectional study	Guangzhou Institute of Respiratory Disease	669 inpatients	Age not specified; 83.4% male
Chen, 2010 (21)	Cross-sectional survey	Friendship Hospital, Beijing	85 inpatients	70.4 years; 72.9% male
Jiang, 2012 (22)	Cross-sectional survey	4th National Health Services Survey (2008) in rural China	13,990 households with a chronic patient	Age/sex not specified
Lou, 2012 (23)	Cross-sectional study	Face-to-face survey, Jiangsu	8,217 patients	61.3 years; 47.8% male
Huang, 2015 (24)	Retrospective observational study	One tertiary hospital, Beijing	1,638 patients	73.4 years; 66.1% male
Wu, 2015 (25)	Cross-sectional study	Community health centers in four cities	678 patients	70.4 years; 72.9% male
Chen, 2016 (26)	Cross-sectional study	Community health centers in four metropolitan cities	678 patients	70.4 years; 72.9% male
Shi, 2018 (27)	Nonrandomized, comparative	Inpatient NCMS database, Henan	6,333 inpatients	67.1–67.6 years; gender not specified
Li, 2018 (28)	Retrospective medical-record analysis	One tertiary hospital, Guangdong	1,943 hospitalized patients	71.2 years; 87.9% male
Chen, 2020 (29)	Retrospective observational study	Twenty hospitals across China	103,510 patients (50,611 inpatients)	Adult patients (≥18 years); gender not specified
Chen, 2020 (30)	Retrospective cohort	Medical claims database from a metropolitan city in south China	11,708 eligible patients	Mainly 65–74; 84% male
Lu, 2022 (31)	Retrospective cohort	NCMS database, Hubei	18,043 high-cost patients	53.6 years; 45.0% male
Cai, 2023 (32)	Repeated cross-sectional surveys	Community-based surveys in three rural counties, Yunnan	954 patients	≥35 years; 49.4% male
Ma, 2023 (33)	Retrospective observational study (Path analysis)	One tertiary general hospital, Ningxia	4,035 patients	Age not specified; 67.4% aged 60–79 years; 66.0% male
Zhao, 2023 (34)	Prospective cohort	COPD-AD China Registry study (single hospital)	579 elderly patients aged >60	68.8 years; 82.6% male
Miao, 2024 (35)	Retrospective observational study	One tertiary hospital, Jiangsu	2,176 elderly (≥65 years) patients	77 years (median); 88.3% male
Xue, 2024 (36)	Interrupted time-series analysis	One tertiary general hospital, Anhui	3,744 patients	Age not specified; 76.3% male in DRG implementation phase
Yan, 2024 (37)	Retrospective observational study	One tertiary comprehensive hospital, Shaanxi	6,748 AECOPD patients	71.14 years (mean); 76.9% male
Dou, 2025 (38)	Cross-sectional study	Nationwide internet-based survey of the urban adult population	361 patients + 361 controls	36% ≥ 60 years; 51.0% male
Gan, 2025 (39)	Retrospective cohort	Urban Employee Basic Medical Insurance database, Tianjin	6,738 patients	68.4 years; 62.3% male
Wang, 2025 (40)	Retrospective cohort	EHRs from 80 hospitals, Tianjin	6,759 patients aged ≥40	68.4 years; 62.4% male
Wu, 2025 (41)	Interrupted time-series analysis	Health insurance database from a pilot city in western China	12,982 inpatients	91.9% ≥ 60 years; 74.3% male
Cheng, 2025 (42)	Retrospective cross-sectional study	Tertiary hospital HIS, Beijing	12,801 hospitalized patients	75.0 years; 69.8% male

AECOPD, acute exacerbation of chronic obstructive pulmonary disease; COPD, chronic obstructive pulmonary disease; DRG, diagnosis-related group; EHR, electronic health record; HIS, hospital information system; NCMS, New Rural Cooperative Medical Scheme; NR, not reported.

### 3.2 Overview of economic burden measures

Definitions of economic burden varied across studies and included direct medical costs, hospitalization expenditures, indirect productivity losses, catastrophic health expenditure (CHE), out-of-pocket (OOP) payments, healthcare utilization costs, and subjective financial burden (Table 2). Despite methodological differences, the included studies generally indicated a substantial patient-level economic burden associated with COPD in China.

**Table 2:** Summary of economic burden measures and key findings.

First author, year	Burden definition	Measurement tool/method	Key quantitative outcome	Main finding regarding burden association
Ren, 2002 (18)	Direct economic burden (annual treatment costs for outpatient care, hospitalization, and medication)	Cost-of-illness method via a one-year prospective survey	In Chengdu, the annual per capita medical cost for COPD patients was ¥1,631.05	The primary factors influencing medical costs were disease severity, quality of life, form of medical expense burden, number of clinic visits, income, and occupation
Chen, 2008 (19)	Hospitalization costs for AECOPD	Retrospective analysis of hospital medical and financial records	The median total hospitalization cost per patient was ¥11,597.6. Drug costs were the largest component, accounting for 71.2% of the total.	Hospitalization cost was significantly correlated with disease severity (lower FEV1%, higher PaCO2), use of mechanical ventilation, and ICU stay
Guo, 2010 (20)	Direct and indirect economic burden	Human capital approach (for indirect costs) and analysis of inpatient expenditure (for direct costs)	The total economic burden was ¥62,844.60 per case, with direct costs accounting for 35.55% (¥22,341.58) and indirect costs for 64.45% (¥40,503.02).	Marital status, length of hospital stay, whether surgery was performed, and patient origin were the main factors influencing the direct economic burden
Chen, 2010 (21)	Subjective financial burden	Patient questionnaire and logistic regression analysis	54.1% of COPD patients reported feeling an economic burden.	The feeling of economic burden was significantly associated with age, income, and sex
Jiang, 2012 (22)	Household Impoverishment & Catastrophic Health Expenditure (CHE)	WHO-recommended capacity-to-pay thresholds	41.9% CHE rate in poorest quintile.	Lower household income was the primary driver of CHE risk.
Lou, 2012 (23)	Direct (medical) and indirect (productivity loss) costs	Custom vulnerability questionnaire; YPLL for indirect costs	Average annual indirect costs (¥20,605) were ~19x higher than direct costs (¥1,090).	83.4% of patients reported stopping treatment for financial reasons.
Huang, 2015 (24)	Direct medical costs (hospitalization expenses)	Analysis of hospital medical record homepage data	The per capita direct economic burden of hospitalized COPD patients increased from ¥19,766.52 to ¥26,133.18 between 2005 and 2013.	The main influencing factors on direct economic burden were age, need for rescue, hospital transference, drug allergy, duration and number of hospitalizations, patient’s condition, discharge outcome, and source of patients
Wu, 2015	Annual direct medical costs	Structured questionnaire and medical records (logistic regression)	Direct medical costs analyzed as a continuous outcome variable	A poorer health-related quality of life (QoL) was significantly associated with higher costs.
Chen, 2016 (26)	Direct (medical and non-medical) and indirect costs of COPD	Patient questionnaires on resource use and costs; Human Capital method for indirect costs	The mean annual direct medical cost per patient was $1,853. Hospitalization accounted for 65.9% of direct costs	The risk of having a high economic burden was 4.68 times greater for patients who had an acute exacerbation compared to those who did not. Age and disease severity were also significant factors
Shi, 2018 (27)	Per-capita inpatient medical expense	NCMS inpatient database analysis (multivariate regression)	Per-capita expense increased, but the effective reimbursement ratio also rose.	The integrated payment policy was associated with higher reimbursement ratios and fewer avoidable readmissions despite rising inpatient expenditures.
Li, 2018 (28)	Hospitalization costs	Analysis of hospital information management system data	The mean total hospitalization cost per admission was ¥24,372.75 ($3,669.33). Western medicine fees were the largest contributor (45.53%).	Longer length of stay, presence of comorbidity, undergoing surgery, antibiotic use, and receiving emergency treatment were all significantly associated with higher total hospitalization costs
Chen, 2020 (29)	Direct medical costs (outpatient and inpatient)	Analysis of hospital information system (HIS) data	Outpatient expenditures were primarily driven by medication costs, whereas inpatient expenditures were dominated by bed and service-related costs.	Inpatient treatment costs include bed fees and other costs, while in outpatient treatment, the main cost incurred is from drugs
Chen, 2020 (30)	Cost of hospitalized acute exacerbations (AECOPD)	Claims database analysis (multivariate regression)	Mean annual AECOPD cost was 38% lower in patients with high medication adherence.	Poor adherence to maintenance medication was associated with higher AECOPD-related costs.
Lu, 2022 (31)	Cost of Potentially Preventable Hospitalizations (PPH)	AHRQ algorithm on ICD-10 codes from claims data	5.8% of inpatient spending for high-cost patients was preventable.	Having COPD was a significant risk factor for experiencing a PPH.
Cai, 2023 (32)	Direct and indirect costs	Structured survey; mean daily income for indirect costs	In 2021, mean total cost was $1,987 per patient.	Higher socioeconomic position was paradoxically associated with higher outpatient costs.
Ma, 2023 (33)	Direct medical costs (hospitalization expenses)	Analysis of hospital medical record homepage data	The average hospitalization cost was ¥17,627.32.	Length of stay is the most direct influencing factor of hospitalization expenses. Other direct factors include admission via outpatient services, whether surgery was performed, and the level of surgery
Zhao, 2023 (34)	Annual total healthcare costs	Claims data analysis using Generalized Linear Models (GLM)	Median annual cost was 37% higher for patients with anxiety/depression.	Comorbid anxiety/depression was associated with substantially higher healthcare costs.
Miao, 2024 (35)	Direct medical costs (hospitalization expenses)	Analysis of hospital medical record homepage data	The average hospitalization cost was ¥18,264.25(range: ¥10,293.60–40,014.66).	Age, length of hospital stay, outcome, admission route, and whether surgery was performed were all significantly associated with hospitalization costs
Xue, 2024 (36)	Direct medical costs (hospitalization expenses)	Analysis of hospital medical record homepage data	After DRG implementation, the average monthly hospitalization cost for COPD patients decreased by 1.82%.	Hospital days, admission status, and medication ratio were the key factors affecting hospitalization costs
Yan, 2024 (37)	Direct medical costs (hospitalization expenses)	Analysis of hospital medical record homepage data	The median hospitalization cost was ¥14,572.81, representing 31.05% of the annual disposable income for urban residents and 83.80% for rural residents in Xi’an.	Hospitalization costs were mainly composed of drug and diagnostic fees. Over the 10-year period, drug costs decreased while diagnostic, service, and nursing fees increased
Dou, 2025 (38)	Direct and indirect costs (work productivity loss)	WPAI questionnaire and self-reported healthcare use	Annual indirect costs ($3,731) were nearly double the direct costs ($1,902).	COPD significantly impaired work productivity vs. matched non-COPD controls.
Gan, 2025 (39)	Clinical and economic burden of AECOPD	Insurance claims data analysis	COPD-related costs for patients with severe AECOPD were 17.8 times higher than for those with no exacerbations	The severity of AECOPD is strongly associated with increased mortality and economic burden in the current and subsequent year
Wang, 2025 (40)	Annual direct medical costs	Electronic health records analysis (GLM)	Annual cost for severe (Group E) patients was 1.7-fold higher than mild (A/B).	Higher disease severity was independently associated with increased costs and mortality.
Wu, 2025 (41)	Economic burden for COPD inpatients (total cost, OOP cost)	Analysis of medical costs per visit from health insurance data	OOP cost per visit decreased by a median of ¥816.93	Comprehensive hospital reform was associated with lower economic burden for COPD inpatients.
Cheng, 2025 (42)	Total hospitalization costs	Retrospective analysis of hospital electronic medical records	The median total hospitalization cost for all patients was ¥21,200.	The presence and type of comorbidity pattern were significantly associated with hospitalization costs. The “cardio-cerebrovascular disease” comorbidity pattern had the highest median cost

Direct medical expenditure, particularly hospitalization costs related to acute exacerbations of COPD (AECOPD), represented the most frequently reported burden component. Median inpatient costs ranged from approximately ¥11,600 to over ¥21,000 per admission (19, 37, 42). Medication costs were a major contributor to expenditure across multiple studies, although more recent studies suggested increasing contributions from diagnostic and service-related fees (19, 37). Disease severity was consistently associated with higher healthcare expenditure. Gan et al. reported that severe AECOPD was associated with COPD-related costs nearly 18 times higher than those in patients without exacerbations (39), while Wang et al. found substantially higher annual costs among patients with more severe disease (40). Longer hospital stay, intensive care admission, mechanical ventilation, emergency treatment, comorbidities, and advanced age were also repeatedly associated with increased hospitalization costs (19, 28, 33, 35, 42).

Several studies highlighted the substantial contribution of indirect economic burden. Productivity loss and disability-related income reduction frequently exceeded direct medical expenditure (20, 23, 38). Lou et al. reported annual indirect loss nearly 19 times higher than direct costs (23), while Dou et al. demonstrated significantly impaired work productivity among COPD patients compared with matched controls (38). Financial hardship also affected treatment continuity, with more than 80% of surveyed patients reporting treatment interruption because of financial constraints (23).

Socioeconomic inequality was a recurring finding. Jiang et al. demonstrated markedly higher CHE rates among lower-income households (22), particularly in rural populations. Similarly, Yan et al. reported that hospitalization costs represented a substantially larger proportion of annual disposable income for rural residents than for urban residents (37). Subjective financial distress was also common, and poorer health-related quality of life was associated with higher direct medical costs (21, 25).

**Figure 2:**
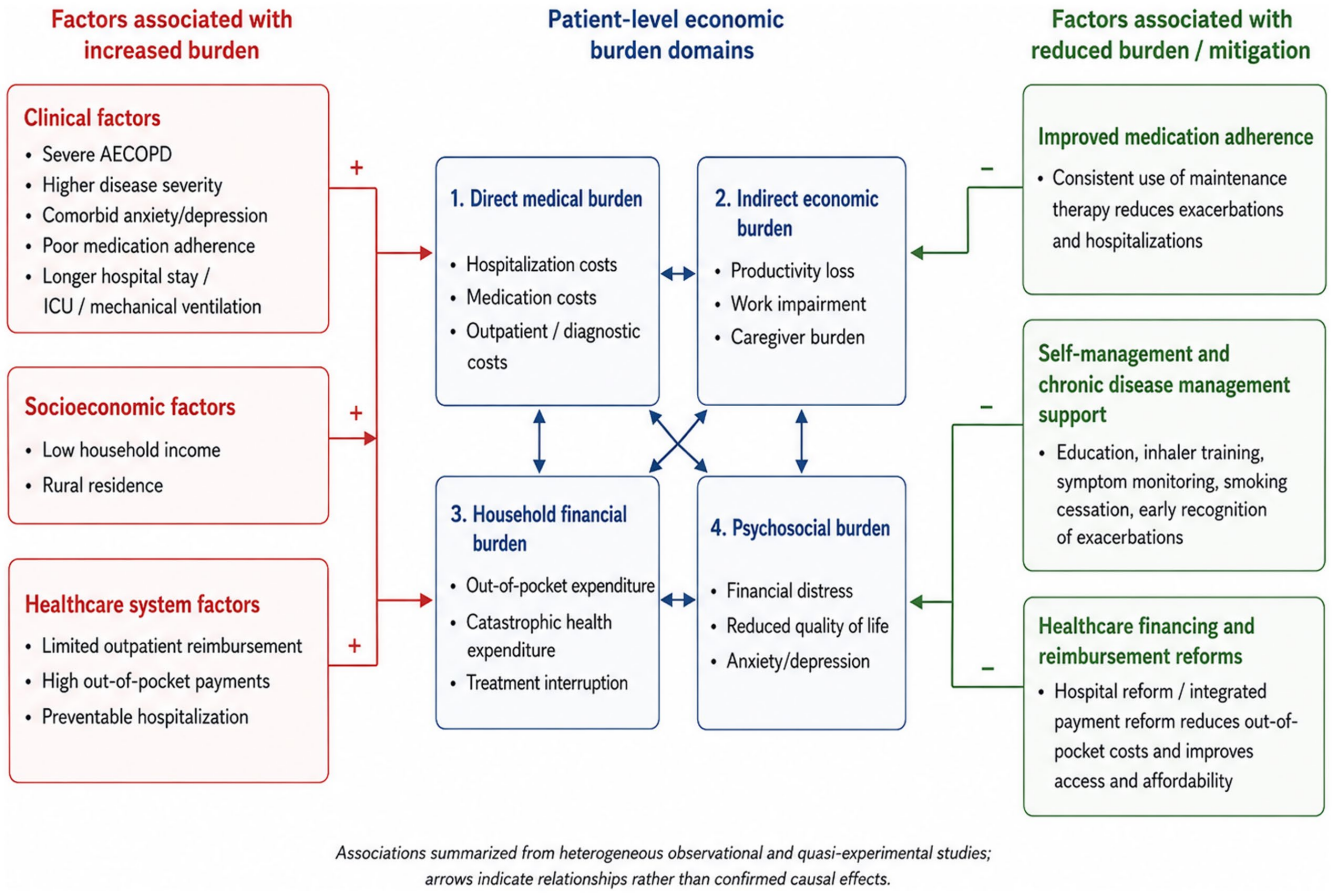
Conceptual framework of factors associated with patient-level economic burden of COPD in China. Summary of factors associated with increased or decreased patient-level economic burden identified in the included studies. The framework illustrates relationships between clinical factors, socioeconomic factors, healthcare financing and service-related factors, and multiple dimensions of economic burden, including direct medical costs, indirect costs, out-of-pocket expenditure, catastrophic health expenditure, and psychosocial burden. The figure summarizes associations reported in heterogeneous observational and quasi-experimental studies and should not be interpreted as demonstrating causality.

Several studies additionally suggested that healthcare financing and reimbursement policies may influence patient-level economic burden. Shi et al. found that integrated payment reform was associated with improved reimbursement ratios and fewer avoidable readmissions (27), while Wu et al. reported lower OOP expenditures after comprehensive hospital reform (41). Lu et al. further identified COPD as an important contributor to potentially preventable hospitalization among high-cost patients (31). These clinical, socioeconomic, and health-system factors and their relationships with different dimensions of patient-level economic burden are summarized in Figure 2.

### 3.3 Methodological quality and risk of Bias

Study-level methodological quality and design-specific risk-of-bias considerations are summarized in Table 3, with overall methodological quality presented in Table 3A and policy-evaluation-specific considerations presented in Table 3B. Overall methodological quality was predominantly rated as fair across the included studies, with five studies rated as good quality and two cross-sectional surveys rated as poor quality. Studies based on administrative databases, insurance claims, and electronic health records generally demonstrated stronger methodological quality because of larger sample sizes, more objective cost measurement, and improved temporal assessment (30, 34, 39–41). Nevertheless, residual confounding and incomplete socioeconomic or clinical information remained important limitations in several retrospective cohort and database studies.

**Table 3:** Methodological quality and design-specific risk-of-bias assessment of included studies.

A. Overall methodological quality assessment of included studies				
Study	Design	Main tool	Key limitations	Global rating
Ren, 2002 (18)	Prospective follow-up survey	NIH	Older study; small community sample; limited reporting	Fair
Chen, 2008 (19)	Retrospective multicenter study	NIH	Hospitalized AECOPD only; hospital-cost focus	Fair
Guo, 2010 (20)	Cross-sectional study	NIH	Inpatient sample; indirect cost estimated by human-capital method	Fair
Chen, 2010 (21)	Cross-sectional survey	NIH	Small sample; subjective burden outcome; self-report	Poor
Jiang, 2012 (22)	Cross-sectional survey	NIH	Chronic-disease household sample, COPD-specific patient data limited; cross-sectional design	Fair
Lou, 2012 (23)	Cross-sectional survey	NIH	Self-reported costs; rural setting; cross-sectional	Poor
Huang, 2015 (24)	Retrospective observational	NIH	Single tertiary hospital; inpatient direct burden only	Fair
Wu, 2015 (25)	Cross-sectional study	NIH	Self-reported and record-based cost data; no temporality	Fair
Chen, 2016 (26)	Cross-sectional study	NIH	Survey-based direct/indirect costs; cross-sectional	Fair
Shi, 2018 (27)	Nonrandomized comparative	NIH + ROBINS-I	Possible confounding and policy/context effects	Fair
Li, 2018 (28)	Retrospective hospital records	NIH	Single tertiary hospital; inpatient costs only	Fair
Chen, 2020 (29)	Retrospective observational study	NIH	Multicenter HIS data; limited patient-level socioeconomic variables; treatment-pattern focus rather than full burden assessment	Fair
Chen, 2020 (30)	Retrospective cohort	NIH	Claims-based adherence proxy; residual confounding	Good
Lu, 2022 (31)	Retrospective cohort	NIH	Claims-based; COPD identified as risk factor in high-cost patients, not exclusively COPD-focused	Fair
Cai, 2023 (32)	Repeated cross-sectional surveys	NIH	Survey-based costs; limited causal inference	Fair
Ma, 2023 (33)	Retrospective path analysis	NIH	Single hospital; path model depends on measured variables	Fair
Zhao, 2023 (34)	Prospective cohort	NIH	Single registry; older patients only; stronger temporality	Good
Miao, 2024 (35)	Retrospective observational	NIH	Elderly inpatients only; single hospital	Fair
Xue, 2024 (36)	ITS/quasi-experimental	NIH + EPOC ITS	Requires pre-trend, autocorrelation, seasonality, concurrent-policy assessment	Fair
Yan, 2024 (37)	Retrospective observational	NIH	AECOPD inpatient cost structure; no causal inference	Fair
Dou, 2025 (38)	Cross-sectional PSM	NIH	Self-reported productivity/costs; PSM reduces but does not remove confounding	Fair
Gan, 2025 (39)	Retrospective cohort	NIH	Claims-based; strong sample and temporal structure; residual confounding possible	Good
Wang, 2025 (40)	Retrospective cohort	NIH	Large EHR database; residual confounding possible	Good
Wu, 2025 (41)	ITS	NIH + EPOC ITS	Needs assessment of pre-trend, autocorrelation, and concurrent policy changes	Good
Cheng, 2025 (42)	Retrospective cross-sectional	NIH	Single hospital; comorbidity pattern analysis, residual confounding	Fair

B. Additional design-specific risk-of-bias considerations for policy-evaluation studies						
Study	Design	Pre-intervention trend reported	Concurrent policy changes addressed	Autocorrelation/seasonality addressed	Outcome measurement reliable	Main methodological concern
Shi, 2018 (27)	Nonrandomized comparative policy evaluation	Not applicable	Partially reported	Not applicable	Yes	Residual confounding and limited control for secular changes
Xue, 2024 (36)	Interrupted time-series analysis	Reported	Partially reported/unclear	Not clearly reported	Yes	Potential concurrent hospital or payment-policy changes
Wu, 2025 (41)	Interrupted time-series analysis	Reported	Partially reported	Partially reported/unclear	Yes	Potential residual confounding from concurrent reforms

EHR, electronic health record; EPOC, Effective Practice and Organisation of Care; HIS, hospital information system; ITS, interrupted time series; NIH, National Institutes of Health; PSM, propensity score matching; ROBINS-I, Risk Of Bias In Non-randomized Studies of Interventions. Global ratings refer to methodological quality rather than certainty of evidence.

Cross-sectional surveys were more frequently limited by reliance on self-reported economic outcomes, subjective financial burden measures, and inability to establish temporal relationships (21, 23, 25, 32, 38). Several hospital-based retrospective studies were additionally restricted to single-center inpatient populations and hospitalization-related expenditures, potentially limiting generalizability to broader COPD populations (24, 28, 33, 35, 42).

For quasi-experimental and policy-evaluation studies, additional EPOC- and ROBINS-I-informed considerations were applied, including pre-intervention trends, concurrent policy changes, autocorrelation or seasonality, and reliability of outcome measurement. The interrupted time-series studies by Xue et al. and Wu et al. both reported pre-intervention trends and used reliable routinely collected outcome data (36, 41), which strengthened internal validity. However, neither study fully addressed potential concurrent policy or healthcare-system changes, and reporting of autocorrelation and seasonality adjustment was incomplete. Similarly, the nonrandomized comparative study by Shi et al. provided useful evidence regarding reimbursement reform but remained susceptible to residual confounding and secular policy effects because of limited control for concurrent system-level changes (27). Despite these limitations, the included studies generally supported the presence of substantial patient-level economic burden associated with COPD across diverse healthcare settings and study populations in China.

## 4 Discussion

This systematic review synthesized evidence from 25 studies published between 2002 and 2025 and identified a substantial, multidimensional patient-level economic burden of COPD in China. Despite heterogeneity in study design and outcome measurement, several consistent patterns emerged. Hospitalization costs related to acute exacerbations represented the principal driver of direct medical expenditure, while indirect costs from productivity loss and work impairment contributed substantially to overall burden. Economic burden was also associated with disease severity, comorbidities, medication adherence, and socioeconomic disadvantage, with lower-income and rural households experiencing disproportionately higher catastrophic health expenditure and out-of-pocket burden. Together, these findings indicate that COPD-related financial burden in China is not limited to healthcare spending, but is closely linked to chronic disease management, insurance design, and health equity.

One of the most consistent findings was the central role of disease severity and acute exacerbations in driving healthcare expenditure. Severe AECOPD, intensive care use, mechanical ventilation, emergency admission, prolonged hospitalization, and comorbidity burden were repeatedly associated with higher inpatient costs. Gan et al. reported nearly 18-fold higher COPD-related costs among patients with severe AECOPD (39), while Wang et al. found higher annual expenditure among patients with more severe GOLD classifications (40). These findings are consistent with international evidence that exacerbation-related hospitalization is a major contributor to COPD costs (43). They also reinforce the economic value of sustained disease control. Chen et al. found that higher adherence to maintenance therapy was associated with lower AECOPD-related hospitalization costs (30), whereas Lou et al. reported frequent treatment interruption because of financial constraints (23). Together, these findings suggest a self-reinforcing cycle in which financial hardship may worsen adherence and disease control, thereby increasing the risk of severe exacerbations and further household costs. Recent evidence suggests that structured COPD self-management interventions, such as inhaler education, symptom monitoring, smoking cessation support, pulmonary rehabilitation, and early exacerbation recognition, may reduce respiratory-related hospital admissions and improve quality of life (44).

The included studies also show that COPD-related burden extends beyond direct medical expenditure. Guo et al., Lou et al., and Dou et al. highlighted substantial indirect costs from productivity loss, disability-related income reduction, and work impairment (20, 23, 38). In some studies, indirect costs exceeded direct medical expenditure, suggesting that healthcare-centered economic evaluations may underestimate the broader household and societal burden of COPD. Psychosocial factors further complicate this burden. Zhao et al. reported higher healthcare expenditure among patients with comorbid anxiety or depression (34), while Chen et al. and Wu et al. linked subjective financial distress and poorer quality of life with greater economic burden (21, 25). These findings indicate that financial stress, mental health, and disease control may be closely intertwined in COPD care.

Socioeconomic inequality was another important finding. Jiang et al. showed higher CHE among lower-income households (22), and Yan et al. reported that hospitalization expenditure accounted for a much larger share of annual disposable income among rural residents than among urban residents (37). These disparities likely reflect differences in income, insurance reimbursement, access to outpatient care, and availability of long-term disease management. Policy-related studies further suggest that financing and reimbursement arrangements may influence patient-level burden. Shi et al. found that integrated payment reform was associated with improved reimbursement ratios and fewer avoidable readmissions (27), while findings from Wu et al. suggest that comprehensive hospital reform may help alleviate OOP expenditure among COPD inpatients (41). However, these quasi-experimental findings should be interpreted cautiously, as residual confounding, concurrent reforms, and incomplete adjustment for secular trends, autocorrelation, or seasonality may remain.

Overall, these findings suggest that stronger outpatient COPD management, adherence support, outpatient reimbursement, and targeted financing policies may help mitigate avoidable economic burden. Priorities include reducing severe AECOPD, improving access to maintenance therapy, strengthening primary care and community-based respiratory services, and protecting lower-income and rural households from catastrophic expenditure. These strategies are particularly relevant for less-developed regions, where delayed diagnosis, limited outpatient reimbursement, and weaker access to specialist respiratory care may intensify the household-level burden of COPD.

This review has several strengths. It incorporated recent evidence up to 2025, including large administrative database studies, multicenter electronic health record analyses, and quasi-experimental policy evaluations. The inclusion of Chinese databases improved retrieval of region-specific literature that may not have been indexed in international databases. The review also considered multiple dimensions of burden, including direct medical costs, indirect costs, CHE, healthcare utilization, and psychosocial burden, thereby providing a broader understanding of COPD-related financial burden in China.

The findings should be interpreted in light of several limitations. Considerable heterogeneity existed across study designs, populations, healthcare settings, and definitions of economic burden, precluding quantitative meta-analysis. Another source of heterogeneity is the long study period covered by the included literature. Studies published between 2002 and 2025 were conducted under different price levels, reimbursement policies, hospital payment systems, and cost-accounting practices. Absolute cost estimates should therefore be compared cautiously, as differences may reflect inflation, regional income growth, insurance reforms, and changes in hospital pricing, as well as variation in COPD severity or healthcare utilization (45). Accordingly, this review emphasized consistent patterns of burden and associated factors rather than direct comparison of monetary values across time periods. Many studies were cross-sectional and partly relied on self-reported expenditure data, increasing susceptibility to recall bias and limiting causal interpretation. Several retrospective studies were restricted to single-center inpatient populations, which may limit generalizability to broader COPD populations. Although interrupted time-series and quasi-experimental studies provided valuable evidence regarding healthcare financing reforms, incomplete adjustment for concurrent policy changes, autocorrelation, and seasonality limited causal inference. Finally, a formal GRADE certainty assessment was not performed because of substantial methodological heterogeneity across the included studies.

## 5 Conclusion

This review shows that the economic burden of COPD in China is driven not only by hospitalization expenditure, but also by productivity loss, socioeconomic inequality, and gaps in long-term disease management. Severe exacerbations consistently emerged as the major contributor to healthcare costs, while lower-income and rural populations faced disproportionately greater financial hardship. The findings suggest that stronger chronic disease management, adherence support, outpatient reimbursement, and targeted financing policies may help mitigate avoidable economic burden among patients with COPD. Future research should prioritize longitudinal and policy-evaluation studies using more standardized economic outcome measures to support the development of cost-effective and equitable COPD management strategies in China.

## Data Availability

The original contributions presented in the study are included in the article/Supplementary material, further inquiries can be directed to the corresponding author.
