# Supplementary material for: The patient-level economic burden of COPD in China: a systematic review of determinants and consequences
Source: Front Public Health. 2026 Jun 29;14:1850249. doi: 10.3389/fpubh.2026.1850249 (PMC13357923; doi:10.3389/fpubh.2026.1850249)
Supplement: Supplementary file 1 [file Supplementary_file_1.docx]

Searching strategy from PubMed

| ( |
| --- |
| ("Pulmonary Disease, Chronic Obstructive"[Mesh] |
| OR COPD[tiab] |
| OR "chronic obstructive pulmonary disease"[tiab] |
| OR "chronic obstructive lung disease"[tiab] |
| OR "chronic obstructive airway disease"[tiab] |
| OR emphysema[tiab] |
| OR "chronic bronchitis"[tiab] |
| OR "airflow obstruction"[tiab] |
| OR "airflow limitation"[tiab]) |
| ) |
|  |
| AND |
|  |
| ( |
| ("Costs and Cost Analysis"[Mesh] |
| OR "Health Expenditures"[Mesh] |
| OR "Cost of Illness"[Mesh] |
| OR "Financing, Personal"[Mesh] |
| OR "Insurance Coverage"[Mesh] |
| OR "Insurance, Health"[Mesh] |
| OR "Reimbursement Mechanisms"[Mesh] |
|  |
| OR "financial burden"[tiab] |
| OR "economic burden"[tiab] |
| OR "financial toxicity"[tiab] |
| OR "financial hardship"[tiab] |
| OR "economic hardship"[tiab] |
| OR "financial distress"[tiab] |
|  |
| OR "catastrophic health expenditure"[tiab] |
| OR "catastrophic expenditure"[tiab] |
| OR "medical impoverishment"[tiab] |
|  |
| OR "out-of-pocket"[tiab] |
| OR "out of pocket"[tiab] |
| OR reimbursement[tiab] |
| OR insurance[tiab] |
|  |
| OR cost[tiab] |
| OR costs[tiab] |
| OR "cost of illness"[tiab] |
| OR expenditure*[tiab] |
| OR expense*[tiab] |
| OR spending[tiab] |
|  |
| OR "hospitalization cost*"[tiab] |
| OR "medical cost*"[tiab] |
| OR "healthcare cost*"[tiab] |
| OR "health care cost*"[tiab] |
|  |
| OR "productivity loss"[tiab] |
| OR "indirect cost*"[tiab] |
| OR "direct medical cost*"[tiab]) |
| ) |
|  |
| AND |
|  |
| ( |
| ("China"[Mesh] |
| OR China[tiab] |
| OR Chinese[tiab]) |
| ) |

Searching strategy from EMBASE

| ( |
| --- |
| ('chronic obstructive lung disease'/exp |
| OR COPD:ti,ab |
| OR 'chronic obstructive pulmonary disease':ti,ab |
| OR 'chronic obstructive lung disease':ti,ab |
| OR 'chronic obstructive airway disease':ti,ab |
| OR emphysema:ti,ab |
| OR 'chronic bronchitis':ti,ab |
| OR 'airflow obstruction':ti,ab |
| OR 'airflow limitation':ti,ab) |
| ) |
|  |
| AND |
|  |
| ( |
| ('health care cost'/exp |
| OR 'health expenditure'/exp |
| OR 'cost of illness'/exp |
| OR 'health insurance'/exp |
| OR reimbursement/exp |
| OR 'out of pocket expense'/exp |
|  |
| OR 'financial burden':ti,ab |
| OR 'economic burden':ti,ab |
| OR 'financial toxicity':ti,ab |
| OR 'financial hardship':ti,ab |
| OR 'economic hardship':ti,ab |
| OR 'financial distress':ti,ab |
|  |
| OR 'catastrophic health expenditure':ti,ab |
| OR 'catastrophic expenditure':ti,ab |
| OR 'medical impoverishment':ti,ab |
|  |
| OR 'out-of-pocket':ti,ab |
| OR 'out of pocket':ti,ab |
| OR reimbursement:ti,ab |
| OR insurance:ti,ab |
|  |
| OR cost:ti,ab |
| OR costs:ti,ab |
| OR 'cost of illness':ti,ab |
| OR expenditure*:ti,ab |
| OR expense*:ti,ab |
| OR spending:ti,ab |
|  |
| OR 'hospitalization cost*':ti,ab |
| OR 'medical cost*':ti,ab |
| OR 'healthcare cost*':ti,ab |
| OR 'health care cost*':ti,ab |
|  |
| OR 'productivity loss':ti,ab |
| OR 'indirect cost*':ti,ab |
| OR 'direct medical cost*':ti,ab) |
| ) |
|  |
| AND |
|  |
| ( |
| ('china'/exp |
| OR China:ti,ab |
| OR Chinese:ti,ab) |
| ) |

Searching strategy from Web of Science

| TS=( |
| --- |
| ( |
| COPD |
| OR "chronic obstructive pulmonary disease" |
| OR "chronic obstructive lung disease" |
| OR "chronic obstructive airway disease" |
| OR emphysema |
| OR "chronic bronchitis" |
| OR "airflow obstruction" |
| OR "airflow limitation" |
| ) |
|  |
| AND |
|  |
| ( |
| financial burden |
| OR "economic burden" |
| OR "financial toxicity" |
| OR "financial hardship" |
| OR "economic hardship" |
| OR "financial distress" |
|  |
| OR "catastrophic health expenditure" |
| OR "catastrophic expenditure" |
| OR "medical impoverishment" |
|  |
| OR "out-of-pocket" |
| OR "out of pocket" |
| OR reimbursement |
| OR insurance |
|  |
| OR cost |
| OR costs |
| OR "cost of illness" |
| OR expenditure* |
| OR expense* |
| OR spending |
|  |
| OR "hospitalization cost*" |
| OR "medical cost*" |
| OR "healthcare cost*" |
| OR "health care cost*" |
|  |
| OR "productivity loss" |
| OR "indirect cost*" |
| OR "direct medical cost*" |
| ) |
|  |
| AND |
|  |
| ( |
| China |
| OR Chinese |
| ) |
| ) |

CNKI

| ("慢性阻塞性肺疾病" OR COPD OR "慢阻肺") |
| --- |
|  |
| AND |
|  |
| ("经济负担" OR "疾病负担" OR "医疗费用" OR "卫生支出" OR "住院费用" |
| OR "灾难性卫生支出" OR "灾难性医疗支出" |
| OR "自付费用" OR "医保" OR "医疗保险" |
| OR "报销" OR "经济压力" OR "财务负担" |
| OR "直接医疗费用" OR "间接费用") |
|  |
| AND |
|  |
| ("中国") |

WanFang

| ("慢性阻塞性肺疾病" OR "慢阻肺" OR COPD) |
| --- |
|  |
| AND |
|  |
| ("经济负担" OR "疾病负担" OR "医疗费用" OR "卫生支出" |
| OR "住院费用" OR "直接医疗费用" OR "间接费用" |
| OR "灾难性卫生支出" OR "灾难性医疗支出" |
| OR "自付费用" OR "财务负担" |
| OR "医保" OR "医疗保险" OR "报销" |
| OR "经济压力") |
|  |
| AND |
|  |
| ("中国") |

Google Scholar

"COPD" OR "chronic obstructive pulmonary disease"

AND

China

AND

("economic burden" OR "financial burden" OR cost OR expenditure OR hospitalization)
